# COVID-19 and the beef market in Latin America: An impact assessment by supply and demand

**DOI:** 10.3389/fpubh.2022.1066168

**Published:** 2022-11-21

**Authors:** Daniela Mejía Tejada, Manuel Francisco Díaz, Karen Johanna Enciso Valencia, Jhon Jairo Junca Paredes, Danny Fernando Sandoval, Stefan Burkart

**Affiliations:** ^1^Universidad EAFIT, Escuela de Economía y Finanzas, Cali, Colombia; ^2^The Alliance of Bioversity International and CIAT, Cali, Colombia

**Keywords:** pandemic, beef consumption, food security, beef production, consumer behavior, resilience, food system, difference-in-differences

## Abstract

The impact of COVID-19 on agricultural markets, especially the beef market, represents one of the greatest food security challenges the world is facing in the post-pandemic era and, for this reason, has been widely documented. This study contributes to the literature through a comprehensive impact analysis of the effects of COVID-19 on both the demand and supply of beef in Latin America and thus provides valuable information for two of the most important links of the beef value chain. Robust econometric methods and a graphic analysis were used that give solidity to the investigation. The analysis used a data panel of supply and demand variables between 2018 and 2022 derived from the US Department of Agriculture. The results suggest that the beef market was strongly affected by the pandemic related health emergency, presenting decreases in both consumption and production. These effects are transitory, however, since the analysis of the post-pandemic data revealed that consumption and production return to normal and seem to grow until smoothing out over time.

## Introduction

The impact of COVID-19 on food security is one of the greatest challenges the world is facing in the post- pandemic era (1–4). Although the indicators of mortality, infections, and hospital occupancy have decreased and the available vaccines facilitated a return to normality, the effects of the economic on the global food systems crisis caused by the implemented public health measures, such as lockdowns, are on the radar of national governments and international organizations as they directly affect the achievement of several of the Sustainable Development Goals (e.g., UN-SDG 2: Zero hunger, UN-SDG 3: Health and well-being, UN-SDG 12: Responsible production and consumption) (5). The Food and Agriculture Organization of the United Nations (6) states that after the first wave of COVID-19 in 2020, around 811 million people suffered from hunger around the world, 118 million more than in 2019, and associates this increase with the impact of the pandemic. Latin America, as a particularly vulnerable region with large numbers of informal employment, is no exception. The consequences perceived after the first wave of COVID-19, together with the mitigation strategies put in place, are among the greatest challenges the region has experienced (7). The FAO (8) further mentions that the pandemic measures in Latin America and the Caribbean, in particular school closures during several months, have caused the suspension of multiple school feeding programs and put food security of approximately 85 million children at risk. The FAO report on the State of Food and Agriculture (9), highlights a special vulnerability of agri-food systems, especially in low- and middle-income countries. It is mentioned that “agriculture is disproportionately exposed and vulnerable to adverse natural hazards (…)”, and “Agrifood systems' components and actors are exposed to shocks and stresses of various types and intensity and, because components are interlinked, disruption in any of them can spread quickly throughout systems” (9). Likewise, the report states that the strongest increase in moderate and severe food insecurity in 2020 was recorded for Latin America and the Caribbean since ~41% of the population suffered from it.in

Hence, it is necessary to carry out rigorous analyses on how and to which extent the food security of, mainly, low- and middle-income countries was affected during the pandemic so that adequate mitigation strategies can be proposed, and a discussion framework generated that helps strengthening the respective sector for future crises.

Within the agri-food system, the cattle sector represents one of the most vulnerable sectors to the effects of a pandemic-type crisis, which is due to the importance the required labor force component. In the case of COVID-19, reductions in the capacity of slaughterhouses and labor shortages due to mobility restrictions represented the greatest challenge (10). In addition, beef is a higher-priced protein source that can be substituted by cheaper options, such as grains, eggs, and chicken. According to FEDEGAN (11) for example, beef was replaced by non-perishable foods such as eggs and rice during the pandemic in Colombia. Despite this, the cattle sector plays a fundamental role within the agricultural sector and the economies of most developing and transition countries and is thus at the center of discussions around food security policy (12–17). In particular, the Latin American cattle sector (i) directly impacts food security as it is an important source of protein (12, 18), (ii) generates direct and indirect employment (19), and (iii) is the major beef producing and exporting region at the global level (12, 13, 20, 21). The largest beef herds in Latin America can be found in Brazil, Argentina, Mexico, and Colombia (16, 22–24).

The public health measures during the first wave of COVID-19 in 2020, particularly lockdowns, mobility restrictions, and the closures of schools and restaurants, abruptly interrupted the production, processing, distribution, and sale of food at both the national and global levels, and have caused an immediate and severe impact on food supply, especially of fresh food such as beef (26b). Likewise, the demand for various commodities, such as beef, has reduced at the same time, which is mainly associated with household income reductions, loss of remittances, or price increases, and the associated substitution effects (2, 25, 26).

Following the thoughts about establishing a discussion framework on how to assure a resilient food system, it is essential to consider the effects on the consumption and production of beef as a case study, since understanding how the food system, and in particular the beef sector, was affected by the pandemic will allow to show trends, observe behaviors, and foresee possible subsequent effects on the aggregate food system.

Although the impacts of COVID-19 on the agriculture and food sector have been extensively studied for specific regions, two gaps can be found in the literature. First, studies on the effects of COVID-19 on consumption are mainly based on perception surveys or self-reported information [e.g., (3, 27–30)]. Although these studies constitute a valuable framework of reference, it is not possible to extract generalizable conclusions from them. Second, the studies tend to focus either on the supply or the demand side as independent markets. In this sense, the present study seeks to contribute to closing these gaps by analyzing the impacts of COVID-19 through a comprehensive impact analysis considering the effects on both beef producers and consumers in Latin America, so that both segments of the beef value chain are covered.

In particular, the study tested two hypotheses related to the beef market and the effects COVID-19 had on it, namely (i) there was a decrease in production associated with the preventive COVID-19 measures, such as lockdowns, school and restaurant closures, and (ii) there was a decrease in demand mainly caused by changes in household incomes of beef consumers. The analysis is focused on three countries including Brazil, Argentina, and Mexico, as well as the South American continent as an aggregate. This analysis is based on the international reference data published by USDA in the USDA Open Data Catalog (31) and used impact assessment techniques such as the estimator of differences over time. This study is structured as follows: Section 2 provides a brief literature review, Section 3 postulates the materials and methods, providing an overview of the data sources and the description of the different models used in the analysis. Section 4 presents a combined section of results and discussion, and finally, Section 5 seeks to draw conclusions and recommendations from the study.

### Literature review

Academia, governmental and non-governmental institutions have devoted much effort to modeling the effects of disturbances in food markets to provide recommendations that help increasing the resilience of agri-food systems. As mentioned in the FAO report on the State of Food and Agriculture (9), a shock of global proportions can come on suddenly, spread rapidly, and compromise the food security, nutritional status, and livelihoods of billions of people to an unprecedented degree and over a long period of time and, as a response to that, it is required to move from commitments to action to transform agri-food systems to make them more efficient, inclusive, resilient, and sustainable.

Within the literature review that relates the effects of COVID-19 with food consumption, two main sources of research were found. First, studies focused on the supply side, i.e., on limitations in supply [e.g., (32–34)], and second, studies focused on the demand side, i.e., on changes in demand and consumer preferences [e.g., (25, 29, 30, 35–37)]. Analyses of the combined effects on supply and demand are rather scarce and occasionally provided rather by non-governmental organizations [e.g., (2)]. In the context of COVID-19, the effects on supply are usually associated with social distancing measures, which cause a sudden limitation of productive capacity, and a drop in the distribution and sale of food, whereas the effects on demand are attributed to several factors, such as reduced household incomes and substitution effects, fear of food shortages, increased food preparation at home, and temporary and permanent closure of restaurants and schools.

The literature review shows that most studies on the effects of COVID-19 on food markets were carried out in the regions or countries mostly affected by the pandemic, namely Asia [e.g., (35)], Europe [e.g., (27–29, 37)], the United States [e.g., (28, 34, 38)]; and Latin America [e.g., (29, 30, 36)]. Regarding research techniques, it is found that the studies are mostly focused on the use of surveys or information collected with representative samples during the most critical months of the pandemic, which provides information on the population interviewed and the reasons for making their decisions. As more robust methods the use of time series and identification of trends was identified as applied by Coluccia et al. (37) who carried out their analysis considering databases and institutional reports from March 2019 to August 2020.

The results obtained in these studies show that the greatest effects were evidenced by abrupt changes in demand. In this sense, two types of behavior were identified: (i) hoarding behavior motivated by fear (29, 35), and (ii) substitution behavior (27, 28, 37). Studies on supply usually conclude that the main problem was that some food did not reach final consumers and was wasted along the different links of the value chains (33).

## Materials and methods

### Data sources and variables used in the study

To examine the impact of COVID-19 on beef consumption and production decisions in Latin America and to be able to observe whether this event indeed corresponds to a discontinuity in the previously evidenced patterns, a data panel was built for the 2018–2022 period. For the construction of this panel, the Latin American countries most relevant at the global level were taken into account, considering their impact on local economies, importance for international trade, and availability of comparable data. In this sense, the following countries were included in this analysis: Brazil, Argentina, Mexico. Additionally, the aggregate of South American countries and the world were included as further elements of analysis.

A series of relevant variables are proposed corresponding to demand and supply effects (37). Table 1 describes the selected independent and dependent variables, their importance, and the consulted sources. All variables were obtained from the international reference data published by the US Department of Agriculture (31). This database indicates the supply, demand, and trade of the main agricultural products for selected countries. These projections provide details of foreign countries supporting the USDA Annual Agricultural Baseline, which are long-range 10-year projections. Hence, the projections used in this document are based on the data and information from the USDA's October 2020 World Agricultural Demand and Supply Estimates (WASDE) to capture the effect of COVID-19 on the beef market (31).

**Table 1:** Dependent and independent variables, importance, sources.

**Variable**	**Definition**	**Importance**	**Source**
**Dependent variables**			
**Demand effects**			
Beef consumption	Beef consumption in 1,000 metric tons	It is the most immediate approximation to the demand for beef and helps to understand if there is a discontinuity caused by the pandemic	(31)
Beef imports	Beef imports in 1,000 metric tons	Although imports do not necessarily reflect consumption patterns, they are a proxy that shows whether the country's demand at a given time could not be supplied by domestic production and had to be supplied by international markets	(31)
**Supply effects**			
Beef production	Beef production in 1,000 metric tons	It is the most immediate approximation to supply of beef as it shows the moment in which producers had to stop/increase their production due to the pandemic	(31)
Beef exports	Beef exports in 1,000 metric tons	Exports reflect international production that was also affected by the lockdowns and effects of the pandemic. In this sense, seeing patterns in the behavior of exports helps to show if the pandemic had an impact at the supply level	(31)
**Independent variables**			
Consumer's price index	Measurement of variation in the price of goods and services representative of household consumption known as the basic basket (39)	This index depends on the basic basket considered in each reference country or region. In this sense, the data used are estimated and projected values	Economic Research Service of the USDA (USDA, n.d.). The sources used by USDA were: International Financial Statistics; International Monetary Fund; IHS Global Insight; Oxford Economics Forecasting
Real Gross Domestic Product per capita	This indicator relates the level of income of a country, measured by the GDP, and its population.	The USDA's Economic Research Service constructed this series from the real GDP series and population data	Economic Research Service of the USDA (31)

### Methodological approach: Estimator of differences over time

Impact assessment is a tool that, through robust techniques and with statistical significance, shows impacts attributable to projects, programs, or specific policies carried out. According to Navarro (40), impact assessment is a summative type evaluation in which it is determined, at the end of an intervention, if it produced the results that were expected during the planning phase.

Among the most recognized impact assessment techniques the difference model, difference-in-difference model, matching methods, discontinuous regression method, and the instrumental variables method can be named (41). The methodology selected for an impact assessment must also be aligned with the type of question that the researcher wants to answer. Considering then the objectives set with this research, it is recognized that the impact assessment seeks to answer a question of cause and effect. In this study the question about which changes the COVID-19 pandemic caused in the beef market for selected Latin American countries. The applied methodology is part of what is known as a natural experiment in which an exogenous factor produces a totally or partially random allocation of treatments (41). As it corresponds to a type of cause-and-effect question, it must be approached considering causal inference, since it must be clearly identifiable to what extent the studied intervention, and only the intervention, changes the initial conditions. Thus, Gertler et al. (42) describe the main formula for impact evaluations of this type, as follows:


$$\begin{array}{l} \propto \;=\;\frac{Y}{P=1}-\frac{Y}{P=0} \end{array}$$

Where the true impact of the intervention (∞) will be given by the results observed at time 1, when the intervention has already been implemented (treatment group), minus the results observed at time 0, where the intervention has not yet been carried out. This is known as the difference-in-difference estimator and corresponds to a specification of the general difference model (41). The difference-in-difference model requires the fulfillment of a strong assumption. For the estimators of the model to be unbiased, that is, not attributing effects to the intervention that do not really correspond to it, it is necessary that the result variables do not show natural trends over time (41). The difference-in-difference model allows estimating the effects of an event (intervention, policy) through a simple regression in which the main regressor is a dichotomous variable (*T*_1_) that takes the value of 1 if the event has already passed (post COVID-19) and takes the value of 0 if the event has not passed yet (before COVID-19).

Since in this study two dependent variables will be considered (supply, demand), two equations have to be estimated, too:


(1) $$\begin{array}{l} \begin{array}{l} BEEF\;CONSUMPTION_{it=\;\beta_{0}\text{+}\beta_{1Imports}\text{+}\beta_{2}T_{1}\text{+}\beta_{3}CPI\text{+}} \end{array}\\ \;\begin{array}{l} \text{+}\beta_{4}GDP\text{\_}PERCA\text{+}U_{i} \end{array} \end{array}$$

(2) $$\begin{array}{l} \begin{array}{l} BEEF\;PRODUCTION{\;}_{it=\;\gamma_{0}\text{+}\gamma_{1}Exports\text{+}\gamma_{2}T_{1}+\;\gamma_{3}CPI\text{+}} \end{array}\\ \;\begin{array}{l} \text{+}\gamma_{4}DGP\text{\_}PERCA\text{+}U_{i} \end{array} \end{array}$$

Where β_2_, γ_2_ represents the estimator of differences for each selected variable and measures the effect of COVID-19 on the associated indicator in the selected countries. As the treatment (having had to face the effects of COVID-19) is randomly assigned, the coefficients will be unbiased. β_*i, i* = 2, 3, 4_, γ_*i, i* = 2, 3, 4_, measure the effects of the macroeconomic variables of the Consumer Price Index (CPI) and Per Capita Gross Domestic Product (GDP_PERCA) for each of the dependent variables.

Furthermore, a graphical analysis of the series considered is provided to illustrate the changes in behavior in the analyzed period, particularly the decreases observed related to the pandemic period and the recovery in subsequent periods.

## Results and discussion

The results presented here constitute a graphic and econometric analysis of the effect of the first wave of the pandemic on the beef market in Latin America. These were critically interpreted and compared to the effects of the COVID-19 pandemic on food markets described in the existing scientific literature, which is constantly growing. Additionally, the assertations of FAO were used overarching observations regarding the situation experienced in the beef market. Table 2 provides information on the descriptive statistics of the analyzed variables. Consumption and production were used as dependent variables and measured in 1,000 metric tons of beef. It is worthwhile to clarify that in the analysis, overall represents the total data set, between represents units (countries), and within represents over time (years).

**Table 2:** Summary statistics of the variables used for the analysis.

**Variable**		**Mean**	**Std. dev**.	**Min**	**Max**	**Obs**
Beef production	Overall	18137.04	27502.58	1,980	73,002	N = 25
	Between		30124.12	2078.4	71710.2	n = 5
	Within		415.613	17125.84	19428.84	T = 5
Beef imports	Overall	2352.848	4346.041	14	11,346	N = 25
	Between		4757.191	15.8	10,849	n = 5
	Within		170.3269	1774.848	2849.848	T = 5
Beef exports	Overall	3049.008	4054.809	272	11,346	N = 25
	Between		4435.334	347.22	10,849	n = 5
	Within		219.0622	2471.008	3546.008	T = 5
Beef consumption	Overall	17440.8	27781.04	1870	73,014	N = 25
	Between		30429.45	1900.24	71709.8	n = 5
	Within		400.3717	16,422	18,745	T = 5
Projected consumer price indices	Overall	193.4512	169.9735	108.87	729.74	N = 25
	Between		163.2957	116.876	485.512	n = 5
	Within		81.66869	−60.8008	437.6792	T = 5
Projected real gross domestic product per capita (in billions USD as of 2015)	Overall	9539.64	1834.237	6766	13046	N = 25
	Between		1944.354	7167.8	12013.6	n = 5
	Within		462.6018	8690.04	10572.04	T = 5

As mentioned above, the changes regarding the beef market in the countries of interest may have occurred to a greater or lesser extent, in the same way, it is possible that the effects occur considering patterns of supply or demand. Thus, the results are presented considering firstly the variables associated with demand (consumption and imports) and subsequently the supply variables (production and exports).

### Changes in the demand for beef

The changes associated with the demand for food and specifically for beef can respond to multiple actions of the human being in extreme situations, such as the declaration of the COVID-19 pandemic in 2020. It is presumed that the changes in demand are mainly associated with fear of food shortages, increased consumption within households and less consumption in restaurants, and sharp and sudden changes in household income (substitution). Therefore, the variables representing variations in demand are: (i) beef consumption, as a direct demand variable and (ii) beef imports as a demand variable covering shortages in the national supply.

Table 3 shows the results of the regression for differences over time considering beef consumption as dependent variable. Initially, 2019–2020 was used as the period of study, which seeks to collect short-term trends, and in a second regression this period was extended to 2018–2022 to collect more permanent effects. These two regressions seek to contrast whether the effects of COVID-19 on the beef market are believed to be transitory or permanent.

**Table 3:** Estimations with beef consumption and beef imports as dependent variable.

	**Short-term effect (2019–2020)**		**Long-term effect (2018–2022)**	
**Variables**	**Beef consumption (with imports)**	**Beef consumption (without imports)**	**Beef consumption (with imports)**	**Beef consumption (without imports)**
D (COVID-19 impact)	−586.543^***^ (0.000)	−1137.383^***^ (0.000)	^**∧**^	722.263^*^ (105.626)
Beef imports	1.851^***^ (0.000)		^**∧**^	
Projected consumer price indices	−1.413^***^ (0.000)	−1.398^***^ (0.000)	^**∧**^	^**∧**^
Projected real gross domestic product (GDP) per capita	−0.535^***^ (0.000)	−0.901^***^ (0.000)	0.216^*^ (0.033)	0.884^**^ (0.066)
Constant	18600.891^***^ (0.000)	26690.113^***^ (0.000)	10512.831^*^ (1,268.003)	8443.327^**^ (546.043)
Observations	10	10	25	25

Robust standard errors in parentheses.

^***^p < 0.01,

^**^p < 0.05,

^*^p < 0.1.

^**∧**^Variables that are not significant at any level of significance.

First, it can be observed that there is a significant difference between beef consumption, measured as beef consumption in 1,000 metric tons for selected countries, before and after the first wave of COVID-19, recalling that the declaration of a health emergency due to COVID-19 occurred in March 2020. Econometrically, this is observed because the variable that measures the impact of COVID-19 (D) is significant and is marking a discontinuity in both specifications. Analyzing the impact of COVID-19 only considering the years 2019 and 2020, the effect is negative and significant. Thus, it is valid to state that the first wave of COVID-19 in 2020 caused a considerable decrease in the consumption of beef.

This result coincides with what was concluded by Peel (38) describing that the effects of COVID-19 on the consumption of beef were transitory and ended toward the end of June 2020. This is because it is presumed that, although it was felt, there was no real shortage of products during the 1st month of closure (lockdown), but bottlenecks were experienced in the supply chains that interrupted the supply processes. Hence, when the bottleneck problems were solved, the supply of supermarkets returned to normal, and consumption quickly stabilized. Peel (38) also highlighted that, although the generalized effects ended quickly, there were economic waves that continued to wreak havoc for longer and that depended on the characteristics of the countries under analysis. Hobbs (43) added that the beef market at the start of the pandemic had to go from a market mostly based in restaurants to a market completely dedicated to supermarkets and food outlets, which required time to adapt (packaging, sanitation requirements, etc.) causing the aforementioned bottlenecks.

**Figure 1:**
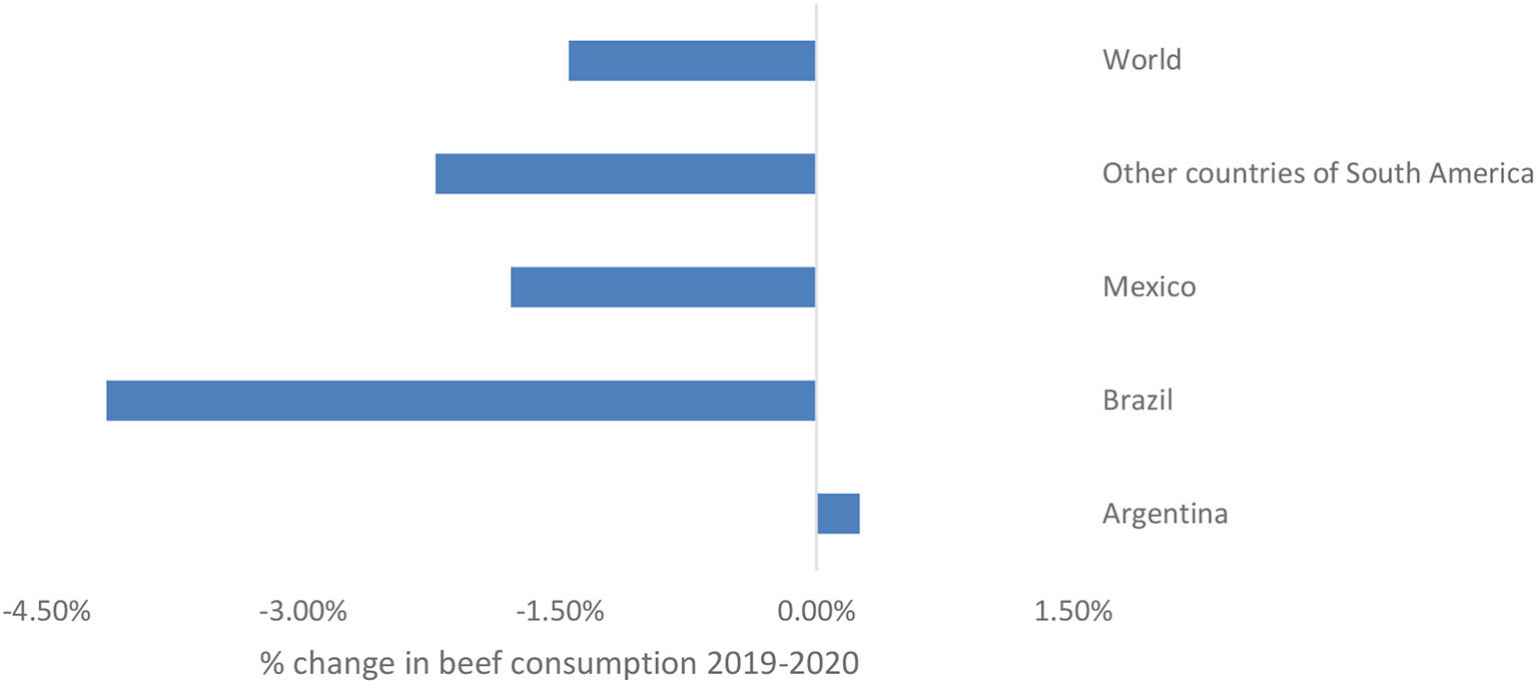
Behavior of beef consumption between 2019 and 2020. Own elaboration based on USDA (n.d.).

It is, therefore, necessary to recognize that the countries selected within Latin America have their particularities and as a result they responded differently to what happened after the declaration of the health emergency. Figure 1 shows the behavioral patterns of beef consumption for the selected countries between 2019 and 2020, illustrating whether it increased or decreased, and to what extent.

It can be observed that the beef market in Argentina indicated a behavior that did not correspond to what was happening in the rest of the world and in the other Latin American countries, since between 2019 and 2020, the consumption of beef increased by 0.25% there, while in other countries from the region it decreased by ~2%, which indicates that the impact of COVID-19 might not have been immediately evident in this particular market. This is in line with information provided by the Institute for the Promotion of Argentine Beef (Instituto para la Promoción de la Carne Vacuna de Argentina, IPCVA) stating that the Argentine beef cattle value chain could react appropriately during the first wave and lockdown, since abrupt changes were not experienced immediately (44). This result can also be contrasted with the monthly economic report of the IPCVA for April 2020, mentioning that the refrigeration industry, both for exports and local consumption, has worked properly and allowed a steady supply within the country throughout the lockdown (45). In Brazil, following Kantar (46), a change in the general consumption habits of Brazilians was evidenced during the first wave, since consumption within the household increased by 27%. Similarly, (47) stated that approximately 18% of Brazilian consumers indicated that they increased their consumption of foods that helped them deal with anxiety, such as ice cream, chocolate, pizza, among other foods. It is worth noting that Brazil showed, like many countries, a reduction in household incomes, high levels of unemployment, and a general fear of the future because of the pandemic, which had a direct and negative impact on monthly spending on food, i.e., of more expensive goods (47). Regarding Mexico, the National Union of Aviculturists (Unión Nacional de Avicultores, UNA) stated that during the pandemic, especially the 1st months, the most important distribution points for the livestock industry were closed. In addition, the lockdown caused the closure of the tourism industry, restaurants, fast food chains, and local markets, which make up approximately 30% of animal protein sales in the country (48).

**Figure 2:**
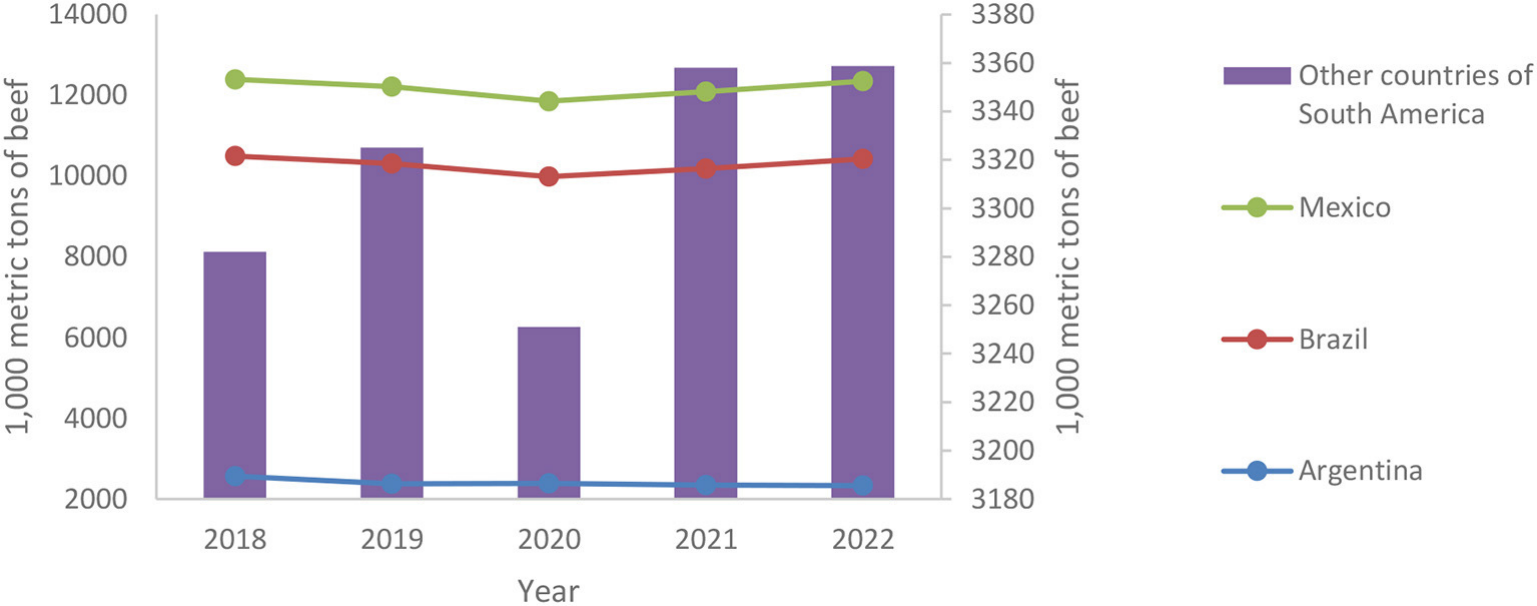
Behavior of beef consumption between 2018 and 2022. Own elaboration based on USDA (n.d.).

The econometric analysis for the long-term period 2018–2022 shows that the effect of COVID-19 on beef consumption is now positive and significant. This is indicating that although COVID-19 caused a general decrease in the immediate consumption of beef during the first wave of the pandemic in 2020, this market recovered in 2021 and the projections reveal that this behavior will continue throughout the year 2022. Figure 2 illustrates the changes in beef consumption for the analyzed countries between 2018 and 2022.

It can be understood that for Latin America the decrease in beef consumption observed between 2019 and 2020, is recovering toward 2021 and 2022. It is observed that the aggregate of the other Latin American countries goes from a drop in beef consumption of 2% between 2019 and 2020 to a growth in of ~3% in 2021–2022, which not only offsets the negative effect experienced by the pandemic, but also evidences an additional increase of consumption. In Brazil and Mexico in particular, the beef market was recovering with growth rates of 3.2 and 2%for 2020 and 2022, respectively. This behavior shows the characteristics of the beef market allowing for a quick recovery, even after suffering from the consequences of crises such as COVID-19. Likewise, it can be observed that beef consumption growth rates are slowing down once pre-pandemic levels (recovery) are reached, indicating that an equilibrium has been found again by now. This coincides with observations made by FAO (6), which state that in Latin America the per capita consumption of animal protein (which already represents 50% of total protein availability in the region) is expected to begin stabilizing or even decline over time.

There are some particularities in the different countries allowing beef consumption patterns to quickly stabilize. For example, for Brazil, Duas Rodas Institucional (47) described that, despite Brazilian consumers wanted to stock up especially on non-perishable and essential foods at the beginning of the pandemic, as the months passed, they began to include non-essential foods such as wines, beer, sweets, and snacks, as well as perishable foods. In Mexico, toward the end of 2020 and as a reaction to the strong effects the pandemic had on the national meat market, the main organizations producing beef, pork, and poultry came together to create Mexico United Animal Protein (México Unido Proteína Animal, MUPA), an initiative that seeks to promote agriculture, industry, and the consumption of domestically produced meat. Similarly, it aims at positioning Mexico internationally as a meat supplier of the highest quality (49).

At the aggregate level of South America, at the end of 2020 at the FAO regional conference for Latin America, three priorities for food system resilience were discussed, namely (i) sustainable food systems, (ii) prosperous and inclusive rural societies, and (iii) sustainable and resilient agriculture. This happened as a reaction to the effects of the pandemic, which exposed the weaknesses of food systems and required restructuring so that they can withstand future crises and abrupt changes more easily and become more resilient. The beef market is an essential part of these food systems since, as mentioned by FAO (6), during the next decade (2021–2030) global animal-sourced protein consumption will increase by 14% (12% in Latin America). and beef consumption by 5.9%, respectively.

**Figure 3:**
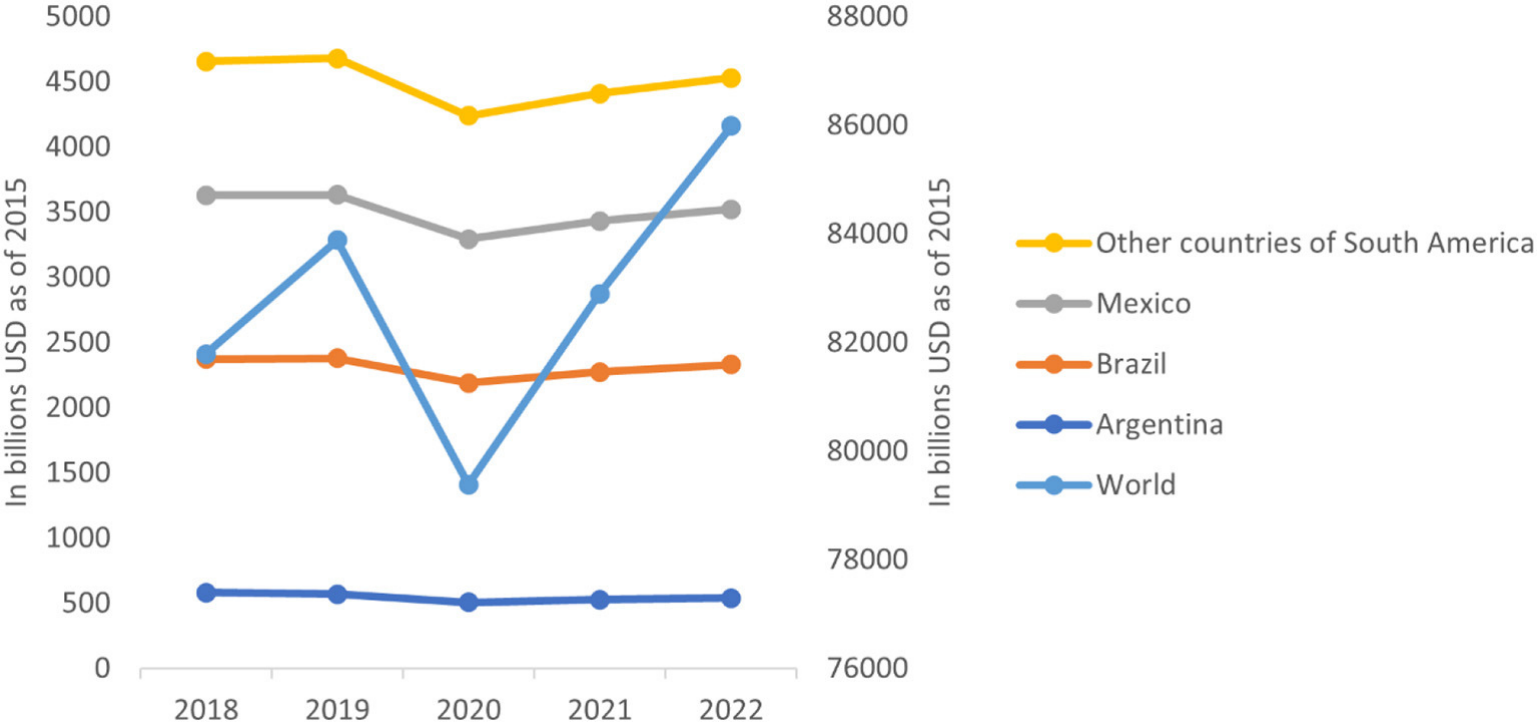
Behavior of the projected real gross domestic product (GDP) between 2018 and 2022. Own elaboration based on USDA (n.d.).

Regarding the other regressors, it is observed that GDP has a positive and significant effect on beef consumption, which is in line with what is expected from the theory, since beef consumption is usually associated with higher levels of wealth and income. Furthermore, it is observed that the relationship between the price index and consumption is negative and only significant considering the first wave, which means that when an increase in prices is perceived immediately due to the pandemic, beef consumption responds by contracting. However, this effect ceases to be significant over time. Figure 3 shows how the GDP behaved within the study period in the different countries and regions. The GDP decrease in 2020 was the most abrupt one recorded for the period of study. In the Latin American countries, the GDP declined by 10% and at the global level by 5.4%, respectively. Although a generalized improvement is visible for the years after the first wave of COVID-19, it can be observed that for Latin America this recovery has occurred gradually, suggesting that the impact of the crisis in the region was of great magnitude. Since beef consumption is associated with higher levels of wealth and income, as it is a rather expensive source of protein, the comparatively strong decline in the GDP for Latin America suggests that the COVID-19 pandemic has caused meat consumers to substitute beef with cheaper protein sources, which is consistent with the results found by other studies [e.g., (25, 26, 30)].

Regarding the effects of the pandemic on the demand of beef, it can be summarized that the first wave of COVID-19 experienced in 2020 caused a discontinuity in the beef market on the demand side, and especially caused lasting effects on beef consumption. Likewise, there was an almost immediate recovery of consumption to pre-pandemic levels after the first wave, which is the result of (i) governmental and marketing strategies that helped the beef market to shift from being almost exclusive to large restaurants toward a positioning in smaller markets, (ii) a felt, but not a real shortage of beef products after the first wave of COVID-19, and (iii) FAO, among other entities, launched recovery programs for food systems allowing countries to have a roadmap to know how to act in this unprecedented situation and minimize the negative effects on their population and food systems. Although it was an unprecedented situation, the beef market, in demand, was able to respond in a resilient way, giving consumers confidence and allowing an almost instantaneous market recovery.

### Changes in the supply of beef

Apart from the effects on the demand for beef, the COVID-19 pandemic may also be associated with changes in the supply of beef since public health measures caused interruptions in beef production and the closure of restaurants, schools, and other establishments, as well as changes in the consumption patterns of society, also could have had effects on beef supply. Therefore, the variables that represent variations in supply are (i) beef production, as a direct supply variable and (ii) beef exports as a supply variable that covers excess production in the national supply.

Table 4 shows the results of the regression for differences over time, considering beef production as the dependent variable. Initially, 2019–2020 was used as the period of study, aiming to collect short-term trends, and in a second regression this period was extended to 2018–2022 to collect more permanent effects. These two regressions seek to contrast whether the effects of COVID-19 on the beef market are believed to be transitory or permanent.

**Table 4:** Estimations with beef supply and beef exports as dependent variable.

	**Short-term effect (2019–2020)**		**Long-term effect (2018–2022)**	
**Variables**	**Beef production (with exports)**	**Beef production (without exports)**	**Beef production (with exports)**	**Beef production (without exports)**
D (COVID-19 impact)	−728.894^***^ (0.000)	−989.181^***^ (0.000)	^**∧**^	895.502^*^ (128.988)
Beef exports	1.425^***^ (0.000)		^**∧**^	
Projected consumer price indices	−1.320^***^ (0.000)	−1.454^***^ (0.000)	^**∧**^	^**∧**^
Projected real gross domestic product (GDP) per capita	−0.607^***^ (0.000)	−0.839^***^ (0.000)	^**∧**^	(0.082)
Constant	20047.529^***^ (0.000)	26739.697^***^ (0.000)	10155.803^*^ (891.658)	8584.818^*^ (675.994)
Observations	10	10	25	25

Robust standard errors in parentheses.

^^***^^p < 0.01, **p < 0.05,

^*^p < 0.1.

^**∧**^Variables that are not significant at any level of significance.

Regarding the beef production variable, measured as beef production values in 1,000 metric tons for the selected countries, it can be observed that the impact of COVID-19 had a significant effect on both specifications. Regarding the first specification, where only the years 2019 and 2020 are considered, we see that the effect of COVID-19 is negative and significant, confirming that in the first wave of the pandemic, where lockdowns and closures occurred most frequently, beef production was affected significantly.

It was found that the effects associated with supply occurred differently from the effects on consumption, specifically they took longer to become evident and were the consequence of market strategies, which is in line with the findings of Peel (38). This delay is strongly related to the first outbreaks of COVID-19 in meat processing plants and slaughtering facilities, which started only around April 2020 and affected workforce availability, caused closures (through public health measures), and thus reduced production levels significantly [e.g., (25, 26, 38, 43, 50)]. As a response to the reduced supply, one market strategy aimed at avoiding supply shortages was to increase beef prices (rationing effect), but this increase was outweighed by disruptions in the value chains which caused a real supply shortage, even though it was temporal, and the sector could recover quickly. This recovery was also due to measures taken by food authorities, allowing temporary exemptions from some food safety and quality standards aimed at accelerating the shift from restaurant sales to sales through food retailers (51).

**Figure 4:**
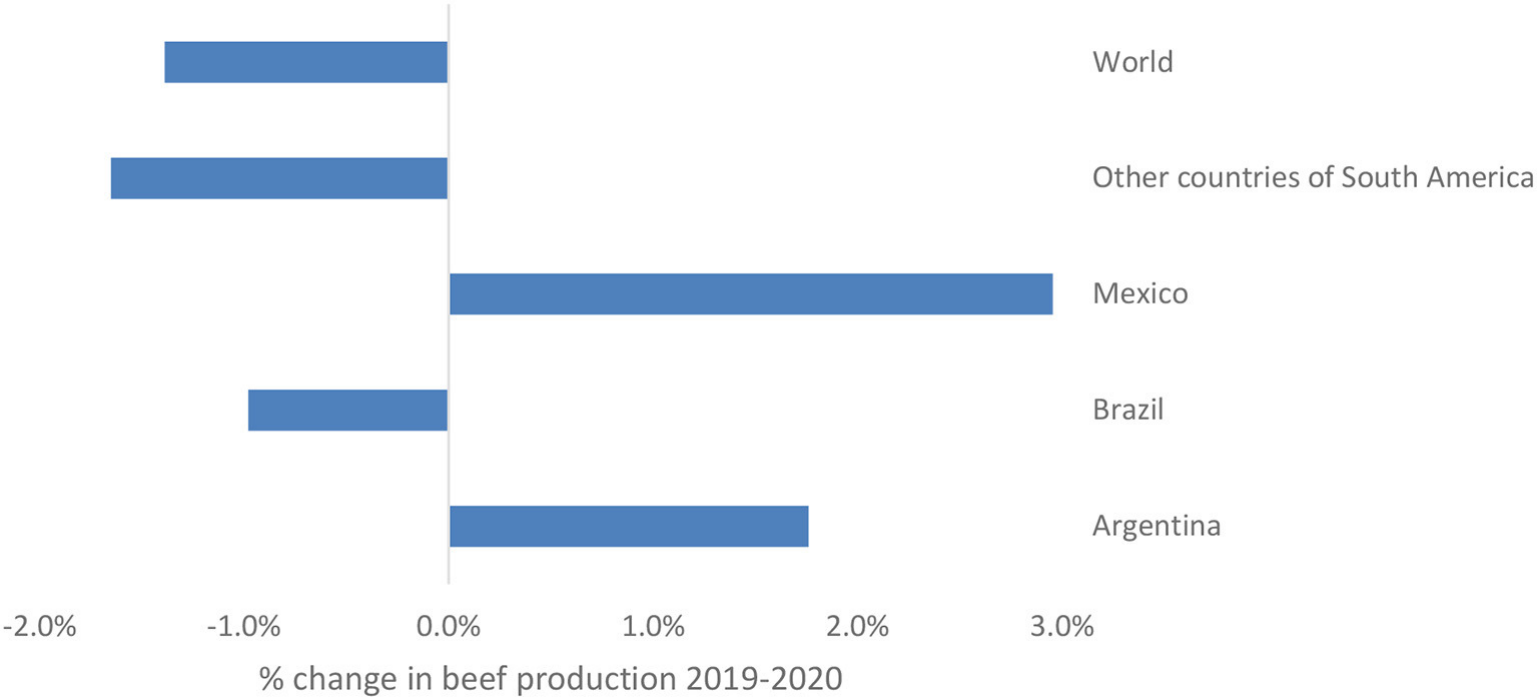
Behavior of beef production between 2019 and 2020. Own elaboration based on USDA (n.d.).

In a similar way as for the demand side, some peculiarities regarding the countries of analysis also occurred on the supply. There were cases in which production was not interrupted immediately. Thus, the effect of the first wave of COVID-19 was not felt between 2019 and 2020, but only by 2021. This was the case for Argentina and Mexico, which had growth rates in beef production by 2020. Figure 4 shows the behavior of beef production between 2019 and 2020, where the first wave of the pandemic occurred. It can be understood that while the world experienced a contraction in beef production by 1.4%, Latin America suffered a decline of ~2% compared to the pre-pandemic levels registered in 2019. Brazil, which is the second largest beef exporter after the United States, experienced a decrease in beef production of 1%, while Mexico and Argentina showed growth rates of 3 and 1.8%, respectively. The particular and contradictory behavior of beef supply in Argentina during the 1st year of the pandemic, is consistent with the observations made by IPCVA (45) who saw this development as a chance to position the country more strongly in the international market. This differentiated behavior also indicates that the beef market behavior is usually particular to the studied area and despite immediate effects of the public measures can be observed, in some countries they took longer to become evident than in others.

**Figure 5:**
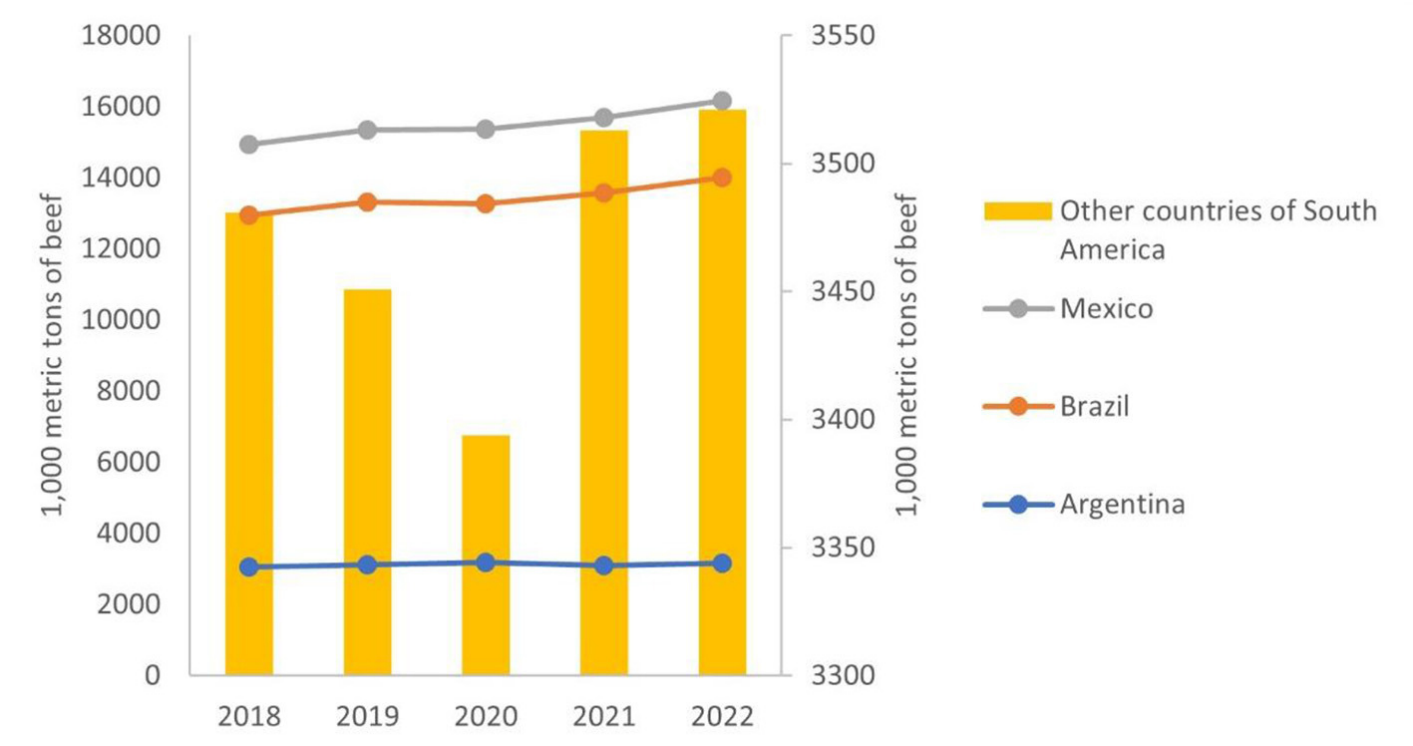
Behavior of beef production between 2018 and 2022. Own elaboration based on USDA (n.d.).

Considering the long-term effects and including the years 2021 and 2022 in the analysis, it becomes visible that the effect of COVID-19 on beef production is still significant, but now positive. This as well as what was observed for beef consumption show that although the initial effect on the beef market was discouraging, it is a market that has managed to recover quickly and that, according to USDA projections, will continue to recover. Figure 5 shows the changes in beef production for the countries analyzed between 2018 and 2022. Data reveals that after the decline in beef production in 2020, an immediate recovery followed at the Latin American level, in Brazil and in Mexico. A behavior that can be evidenced at a general level is that beef production seems to stabilize over time which coincides with what was described for beef consumption as well.

**Figure 6:**
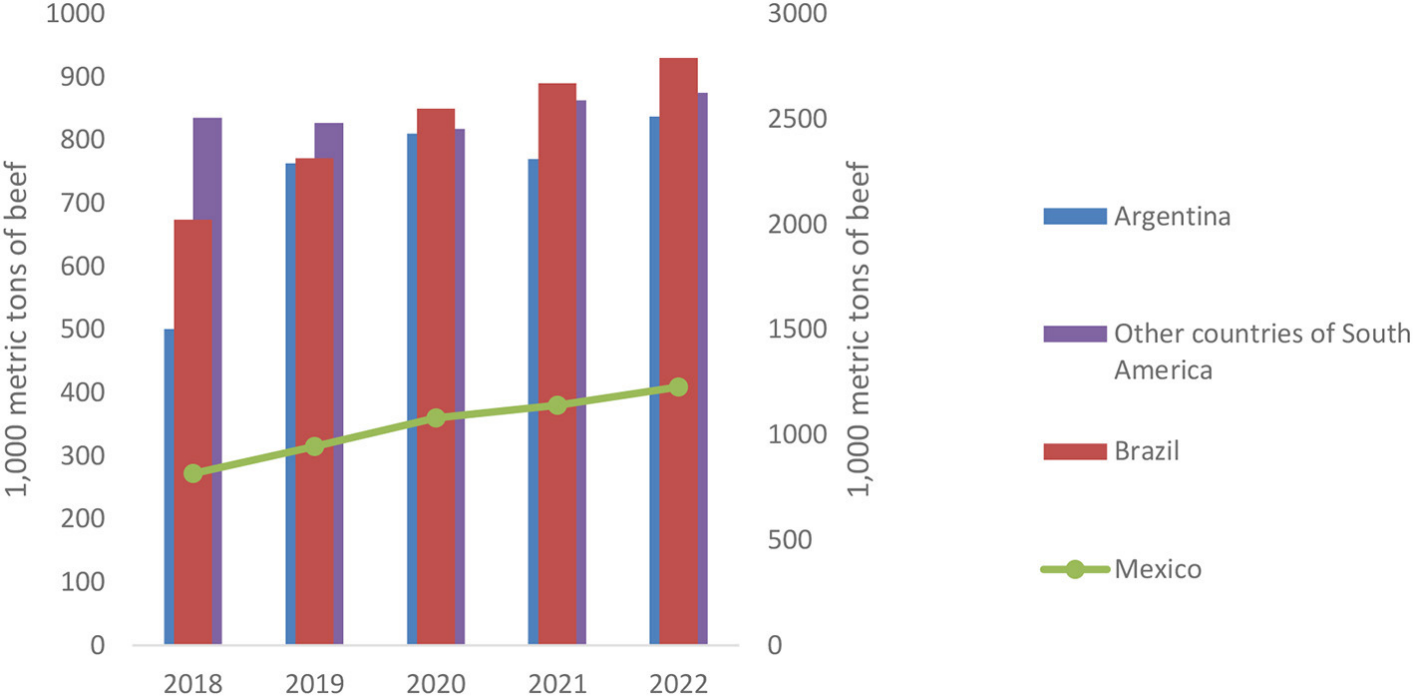
Behavior of beef exports between 2018 and 2022. Own elaboration based on USDA (n.d).

Beef exports, as a measure of international trade, had a significantly positive development during the first wave of the pandemic in 2020 (short-term) and a positive, but not significant development thereafter (long-term). The graphical analysis in Figure 6 indicates that this positive trend is kept over time. In other words, the effects of COVID-19 caused an increase in beef exports that is expected to be sustained considering the USDA projections for 2022. This increase may be associated with the closures occurring in different countries and with the restrictions surrounding production. It might be possible that some countries had to start importing beef and countries like Brazil, a traditional beef exporter, could take advantage and increase their export volumes. This data also evidences the strong importance of the beef market, and especially the exports, in the analyzed Latin American, since Brazil shows export growth rates throughout the study period, Argentina illustrates a decline of 5% in 2021, but manages to fully recover toward 2022 with a growth rate of 9%, and Mexico increased exports by 14% in 2020 and 6% in 2021, respectively. The findings are in line with what IPCVA (45) reported for Argentina: the decline in exports in 2020 was due to (i) the complete closure of the HORECA complex (hotels, restaurants, and catering) in the United States, which caused the temporary suspension of some already agreed shipments, and (ii) the interrupted free trade with European countries (45). Later, in 2021, Argentine beef exports reached record export volumes, especially with the consolidation of China as the main customer (52).

Taking the macroeconomic indices into consideration, it can be observed that the relationship between beef production and GDP is negative if transitory effects are evaluated and positive when permanent effects are considered. Similar to beef consumption, this relationship manages to show that the beef market is associated with wealth and income. Regarding the consumer price index, a negative relationship is observed which is only significant when considering the effects of the first wave. This is evidenced in Figure 3 illustrating the effects that COVID-19 had on the GDP.

As documented for the demand side, the first wave of COVID-19 in 2020 caused a discontinuity in the beef market on the supply side, too. Lasting effects are visible in both beef production and beef exports. It is worth mentioning that the supply and demand sides are complementary, indicating that some mitigation strategies taken on one side also influence the other side. For example, strategies aimed at lowering food safety standards to support the supply side also influence decisions on the demand side.

## Conclusions and recommendations

This study seeks to show empirical and robust results on the effects of COVID-19 on the production and consumption, as well as the exports and imports of beef in Latin America. It is, thus, a contribution to the existing empirical evidence on the vulnerability of the cattle sector and food systems in general to crises or unprecedented shocks. The results thus help policy makers in taking measures to increase the resilience of the sector and prepare food systems better for future crises.

As the effects of COVID-19 on the different spheres of human behavior have been extensively studied throughout the world, the brief literature review provided in this study revealed that there are effects associated only with demand and only with supply, or combined effects when it comes to food consumption that can jeopardize the food security of the most vulnerable countries. Additionally, the presented research showed that the effects COVID-19 had on the beef market in Latin America, both considering the demand and supply sides, seem to be of temporary nature and recovery has happened quickly, indicating a high level of resilience to external shocks.

The results revealed in this document are based on the compilation of different global databases provided by USDA and may vary from the data reported by each country or region in their respective official sources. The estimates provide information showing that COVID-19 had a negative effect on the beef market of a transitory type (2019–2020), which reversed thereafter and is now positive (2021–2022). It was shown that the discontinuity caused by the pandemic decreased the consumption and immediate production of beef.

The econometric results presented in this study presume that the recovery in consumption was rapid since (i) there was no real shortage of products during the 1st month of closure, but rather bottlenecks were experienced in the supply chains that interrupted the supply processes, (ii) beef consumption moved from the social sphere (restaurants) to the private sphere (households), and (iii) there was a transversal accompaniment by entities such as FAO helping the countries in establishing roadmaps focused on mitigating the situation. These results are consistent with findings documented in the literature. Regarding beef supply, the results of this study are contrary to the findings for the demand side, since beef production suffered a contraction at the beginning of the pandemic due to outbreaks and temporary closures of production facilities, followed by rationing effects to avoid shortages that caused increases in beef prices, and finally measures adopted by the food authorities that allowed temporary exemptions from quality standards to accelerate market recovery. The results of this study document an almost cohesive and instantaneous behavior that allowed the prompt recovery of the beef sector in the analyzed markets.

Similarly, it was found that the beef market growth rates were lower in 2022 than in 2021. This might be related to general shifts in consumption (as documented by FAO or USDA) toward other (cheaper or healthier) protein sources, more sustainable products, or plant-based diets. It is recommended that the beef market evaluates and reacts to these cultural changes so that the shares of beef in consumer diets can be maintained over time. The results presented in this study along with the provided discussion allow to draw policy recommendations regarding the beef market in the analyzed countries. It became evident that the cattle industry is of paramount importance for the economies and the diets in Latin America, since it corresponds to a large part of the animal-sourced food intake in these countries and therefore strongly contributes to food security. Considering this significance, any market disturbance hitting this industry, such as COVID-19, also affects the welfare level of the households. Therefore, it is recommended that the beef industry starts incorporating such disturbances in their market projections and takes actions to increase resilience of the sector. Potential actions for increasing resilience exist, for example, in the development of differentiated beef products that consider e.g., environmental, social, and animal welfare attributes (26), and in the sustainable intensification of the cattle sector (53).

This document also provides information on the use of empirical strategies that respond to impact evaluations related to exogenous events such as COVID-19, generating discontinuities in the relevant variables for the beef market. Finally, the objective of showing different alternatives to model these relationships and provide more robust information for decision makers is fulfilled.

## Limitations of this study

The models used in this study and the database imply a series of limitations when making assertions with the results. The differences-in-differences model assumes the fulfillment of several assumptions that are strong and that, if not fulfilled, may be yielding biased estimators. It assumes that for the estimator to be effectively unbiased and to show the effect only of the intervention being evaluated, there must not be a time trend in the outcome variable, that is, the outcome variable cannot have a clear trend over time that can be confused with the results of the intervention. Bernal and Peña (41) explain that if there is a way to have more than two observations over time for each individual, it is possible to model this trend and mitigate the risk of having biased coefficients.

Regarding the data collected by USDA used in this study, there are some drawbacks. In the first place, the data corresponding to 2021 and 2022 are estimates made by the USDA Economic Research Service, which, despite providing information based on economic support data, is still a projection that may or may not adhere to reality. In addition, these data are collected from different international sources which can cause differences from country to country.

Another problem that stems from working with estimates is that since the first wave of COVID-19 in 2020, not enough years have passed yet to allow for performing robustness tests on the models and by this guaranteeing that the effects observed for the beef market can be attributed solely and exclusively to COVID-19.

Taking these limitations into account and the way in which the document seeks to recognize and mitigate them, it is considered that the results obtained are valid and contribute to the literature that seeks to model the effects of COVID-19 on food security.

## Data availability statement

Publicly available datasets were analyzed in this study. This data can be found here: https://www.usda.gov/topics/data.

## Author contributions

DM, KE, MD, and SB: conceptualization and resources. DM, KE, JJ, and SB: methodology. DM, KE, JJ, and DS: formal analysis. DM, JJ, DS, and SB: writing the original draft and review and editing. SB: supervision, funding acquisition, and project administration. All authors contributed to the article and approved the submitted version.

## Funding

This work was funded by the CGIAR Research Program on Livestock. In addition, it was supported by the OneCGIAR Initiative on Livestock and Climate (L&C). The funders had no role in the design of the study; in the collection, analyses, or interpretation of data; in the writing of the manuscript, or in the decision to publish the results.

## Conflict of interest

The authors declare that the research was conducted in the absence of any commercial or financial relationships that could be construed as a potential conflict of interest.

## Publisher's note

All claims expressed in this article are solely those of the authors and do not necessarily represent those of their affiliated organizations, or those of the publisher, the editors and the reviewers. Any product that may be evaluated in this article, or claim that may be made by its manufacturer, is not guaranteed or endorsed by the publisher.

## Author disclaimer

The views expressed in this document may not be taken as the official views of these organizations.
